# Pre-treatment pathogen testing rates before antimicrobial therapy in emergency departments: a four-year retrospective study from two hospital campuses

**DOI:** 10.3389/fcimb.2026.1730665

**Published:** 2026-06-29

**Authors:** Suling Huang, Wanting Lu, Yafang Hong, Jingfa Zhu, Yusheng Liu, Zhijun Su

**Affiliations:** Quanzhou First Hospital Affiliated to Fujian Medical University, Quanzhou, Fujian, China

**Keywords:** antibiotic resistance, antimicrobial stewardship, emergency department, empirical antibiotic therapy, microbial culture, pathogen testing rate

## Abstract

**Background:**

To monitor and analyze the current status and trends in pretreatment pathogen testing rates for antimicrobial therapy in the emergency departments of two campuses of a tertiary hospital, explore key factors influencing pathogen testing rate, and propose improvement strategies.

**Methods:**

Data from 534,374 visits from the emergency departments between 2021 and 2024 were collected via the hospital information system, outpatient logs, and laboratory management system. Antibiotic utilization rates and pretreatment pathogen testing rates were calculated. Statistical analyses included chi-square tests, Cochran-Armitage trend tests, Cramer’s V, and logistic regression.

**Results:**

Over four years, antimicrobial usage increased from 17.95% to 22.59% (Z = 41.79, P < 0.001), whereas the pathogen testing rate remained consistently low at 13.08%–14.17%, with no improvement (Z=−1.70, P = 0.089). The pathogen testing rate at Dongjie Campus was significantly lower than that at East City Campus in all years (P < 0.001). From 2021 to 2023, the pathogen testing rate in internal medicine departments exceeded that in surgical departments, but this trend reversed for the first time in 2024 (internal medicine: 12.42% vs. surgery: 13.86%, P<0.001). Procalcitonin was the primary specimen type, with its proportion declining annually; Mycoplasma pneumoniae testing showed rapid growth; and traditional bacterial culture consistently accounted for less than 5% of the samples.

**Conclusion:**

Emergency antimicrobial usage rates have persistently increased, whereas pathogen testing rates have remained chronically low, revealing a prominent contradiction of “high usage, low submission.” Significant variations exist across campuses and departments. The submission structure is imbalanced, with severe inadequacies in microbial culture. We recommend establishing a multidisciplinary collaborative management model, strengthening information-based interventions, optimizing submission workflows, and implementing long-term oversight and incentive mechanisms to achieve targeted antimicrobial therapy and antimicrobial resistance control objectives.

## Introduction

Antimicrobial resistance (AMR) has become a global public health challenge, severely threatening the ability of modern medicine to treat infectious diseases (Aggarwal et al., 2024, (Adhikari et al., 2025). As AMR levels rise, the associated mortality rates and global economic burden are projected to increase accordingly (Antimicrobial Resistance Collaborators, 2022). The findings of the UK Government-commissioned AMR Review indicate that by 2050, AMR could cause 10,000,000 deaths annually (compared with 8.2 million annual deaths from cancer) (). The World Health Organization (WHO) has repeatedly called on countries to place antimicrobial stewardship (AMS) at the core of national health policies and to develop globally coordinated action plans to collectively address this urgent challenge (Prestinaci et al., 2015, Mendelson and Matsoso, 2015).

The misuse of antimicrobial drugs is widely recognized as a major driver of increasing AMR (Sweileh, 2021), and optimizing antimicrobial use is a critical measure for controlling bacterial resistance (Shallcross and Davies, 2014, Li et al., 2022).The emergency department is an important setting for the use of antimicrobial drugs. However, in the high-intensity, fast-paced environment of emergency departments, clinicians frequently face challenges of high diagnostic uncertainty and significant time pressure, making empirical antibiotic prescribing a common choice. A nationwide database study in China further indicated that up to 62.0% of antimicrobial prescriptions in emergency department visits were deemed inappropriate (Zhao et al., 2021). However, medication decisions lacking supporting etiological evidence may exacerbate the development of bacterial resistance. Studies indicate that approximately one-third of patients receiving broad-spectrum antimicrobial therapy in emergency departments are ultimately diagnosed with noninfectious or viral conditions (Newson et al., 2022, Shappell et al., 2021). Pathogen testing serves as a bridge connecting empirical treatment and targeted treatment. The rate of pathogen testing before antimicrobial treatment for emergency patients (hereinafter referred to as “pathogen testing rate”) has become one of the core quality indicators for measuring the level of rational drug use in medical institutions and the effectiveness of antimicrobial stewardship (AMS).

Despite the explicit requirements to strengthen pathogen testing outlined in the National Health Commission’s “Administrative Measures for the Clinical Application of Antimicrobial Agents” and multiple guidelines (National Health Commission of the People's Republic of China, Bureau of Medical Administration and Medical Service Management, 2021) (National Institute of Hospital Administration, National Health Commission of the People’s Republic of China, 2021, National Health Commission of the People's Republic of China, Bureau of Medical Administration and Medical Service Management, 2022), pathogen testing rates in emergency departments across most domestic hospitals remain unsatisfactory, with significant regional and interhospital disparities (Zachrison et al., 2025). Current research on antimicrobial testing rates predominantly focuses on hospital-wide cross-sectional surveys or macrolevel policy analysis, with insufficient in-depth exploration of the complex emergency department setting. Most studies merely describe the phenomenon of low pathogen testing rates without thoroughly analyzing the underlying multilayered, systemic factors, such as barriers in clinical pathways, time constraints in clinical practice, differences in healthcare provider awareness, and the support capacity of laboratory departments. The two campuses of a tertiary hospital selected in this study exhibit significant differences in hardware facilities, patient populations treated, and management and operation models, providing a valuable natural control for examining the effectiveness of diagnostic management measures for antimicrobial agents in different actual medical environments.

This study aimed to systematically monitor preantimicrobial pathogen testing data among emergency patients at two campuses of a tertiary hospital from 2021--2024. This study aims to analyze the current pathogen testing rate and its influencing factors thoroughly while exploring effective intervention strategies to increase pathogen testing rates. This research endeavors to provide data-driven support and practical guidance for the scientific management of antimicrobial agents in emergency departments.2 Subjects and methods.

## Methods

### Study subjects

A total of 534,374 visits from the emergency departments (ED) of two campuses of a Grade III Class A hospital between 2021 and 2024 were included in this retrospective analysis. The analysis was performed at the visit level rather than the patient level. Therefore, if the same patient presented to the ED multiple times during the study period, each eligible visit was counted separately, provided that it had a unique visit record and met the study criteria. These visits were categorized into emergency internal medicine and emergency surgery groups. Both initial and follow-up ED visits were included. Pediatric ED visits, obstetric/gynecology ED visits, and inpatient cases were excluded.

### Data collection

Data were extracted from the hospital information system, outpatient logs, and Ruimei Laboratory Management System. The outpatient visit information included the registration time, patient ID number, number of visits, patient name, registration department, and prescribing physician. The antimicrobial usage details included the patient ID number, number of visits, patient name, antimicrobial name, prescription date, confirmation date, and administration route. Prescription-related microbiology testing items (item names included culture, routine tests, Legionella pneumophila IgM antibody, Neisseria gonorrheae, Chlamydia pneumoniae IgM antibody, Mycoplasma pneumoniae IgM antibody, spirochetes, TPPA, fungi, D-glucan, RPR, Cryptococcus, etc.) and inflammatory biomarkers (Procalcitonin, interleukin-6) (**Table 1**).

**Table 1:** Model specification table.

Item	Specification
Dependent variable	Pre-antibiotic microbiology testing among antibiotic-treated ED visits (1 = tested; 0 = not tested)
Binomial response for aggregated data	cbind(tested, antibiotic-treated visits - tested)
Main predictor	Clinical group: Surgery vs Internal Medicine
Reference category for clinical group	Internal Medicine
Covariates	Calendar year and campus
Reference categories for covariates	Year 2021 and East campus
Primary model formula	cbind(tested, not tested) ~ group + factor(year) + campus
Reported effect estimate	Adjusted odds ratio (aOR), 95% confidence interval, and P value

### Traditional bacterial culture submission indicators

In addition to the overall pre-antibiotic microbiology testing rate, we separately calculated traditional bacterial culture submission rates per antibiotic-treated ED visit. Traditional bacterial cultures included blood culture, routine bacterial culture, and urine culture. The submission rate was calculated as the number of culture submissions divided by the number of antibiotic-treated ED visits. Because the available data were aggregated at the specimen/order level, the combined traditional bacterial culture submission rate should be interpreted as culture submission intensity per antibiotic-treated ED visit rather than a mutually exclusive patient-level culture rate.

### Preantimicrobial therapy pathogen testing rate

The pre-antimicrobial therapy pathogen testing rate was calculated as follows: (number of ED visits in which pathogen-related testing was performed before therapeutic antimicrobial use within a specified period/number of ED visits receiving therapeutic antimicrobial agents during the same period) × 100%.

### Logistic regression model specification

To compare the likelihood of pre-antibiotic microbiology testing between clinical groups, we fitted a binomial logistic regression model among antibiotic-treated ED visits. The dependent variable was pre-antibiotic microbiology testing status, defined as 1 if a microbiology test was ordered before antibiotic administration and 0 otherwise. The main predictor was clinical group, with Surgery compared against Internal Medicine. Internal Medicine was used as the reference category. Calendar year and campus were included as covariates, with 2021 and the East campus used as reference categories. Because the available dataset was aggregated by campus, year, clinical group, and physician, the model was fitted using a binomial response of the form cbind(tested, not tested), where tested was the number of patients with a pre-antibiotic microbiology test order and not tested was the number of antibiotic-treated patients without such an order. The model was specified as follows: logit{P(Y = 1)} = *β_0_* + *β_1_*(Surgery) + *β_2_*(Year_2022)_ + *β_3_*(Year_2023_) + *β_4_*(Year_2024_) + *β_5_*(Cheng campus).

where Y = 1 denotes pre-antibiotic microbiology testing. Adjusted odds ratios (aORs) and 95% confidence intervals were reported. In annual stratified analyses, Surgery and Internal Medicine were compared within each calendar year using 2 x 2 contingency tables, and chi-square statistics, P values, and Cramer’s V were reported.

### Statistical analysis

All the data were organized and analyzed via R software version 4.3.1. Count data are expressed as counts (percentages). Comparisons of dichotomous or categorical variables were performed via the chi-square test. To assess trends in antimicrobial usage rates and pathogen testing rates over time, the Cochran–Armitage test for trend was employed to calculate trend Z values and corresponding P values. Cramer’s V was used to measure the effect size of the intergroup differences, ranging from 0 to 1, with higher values indicating stronger associations. The 95% confidence interval (95% CI) for the pathogen testing rate was calculated via the Wilson score interval. The odds ratio (OR) and its 95% CI for surgery versus internal medicine were estimated via a logistic regression model and are presented on a logarithmic axis. A two-tailed P value < 0.05 was considered statistically significant.

## Results

### Antimicrobial use and pretreatment pathogen testing among emergency patients across two hospital campuses

**Figure 1:**
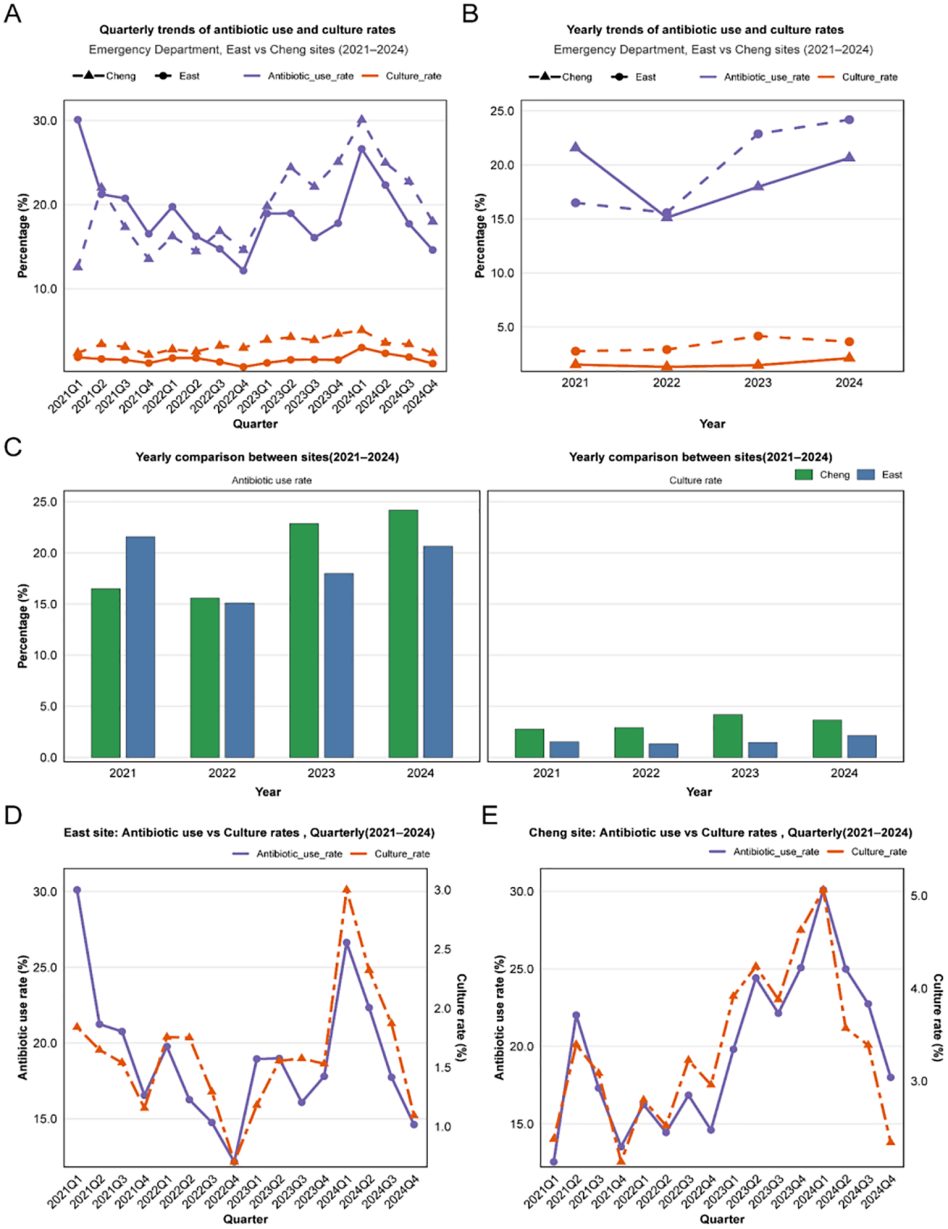
Antibiotic prescribing and microbiology culture testing in the emergency department at two sites (East vs Cheng), 2021–2024. **(A)** Quarterly trends in the antibiotic use rate and pathogen testing rate. Points denote sites (East vs Cheng); lines denote the two indicators. **(B)** Yearly trends (totals by year). **(C)** Yearly comparison between the two sites; bars show rates by year, faceted by indicator (left: antibiotic use rate; right: pathogen testing rate). **(D–E)** Dual-axis quarterly plots for each site (left y-axis: antibiotic use rate; right y-axis: pathogen testing rate).

From 2021 to 2024, a total of 534,374 ED visits were enrolled, including 225,358 at Campus One and 309,016 at Campus Two. The overall antimicrobial usage rate increased annually, increasing from 17.95% (20,089/111,907) in 2021 to 22.59% (35,001/154,918) in 2024. The Cochran-Armitage trend test yielded Z = 41.79, P < 0.001; Cramer’s V = 0.071, indicating a significant but small effect size. The pathogen testing rate fluctuated between 13.08% and 14.17% over the four years, remaining at a low level with no clear upward trend (Z = −1.70, P = 0.089) (See **Table 2**; **Figure 1**).

**Table 2:** Antimicrobial use and pretreatment pathogen testing among emergency patients, 2021–2024 (combined data from both hospital campuses).

Year	Number of visits	Number of antibiotic users	Antibiotic use rate (%)	Number of microbiology tests	Pathogen testing rate(%)
2021	111,907	20,089	17.95	2,690	13.39
2022	133,648	20,539	15.37	2,911	14.17
2023	133,901	27,559	20.58	3,882	14.09
2024	154,918	35,001	22.59	4,577	13.08
χ² (df=3)	—	—	2676.183	—	20.099
P (χ²)	—	—	<0.001	—	0.00016
Z	—	—	41.79	—	−1.70
P (Trend)	—	—	<1×10^-^¹^6^	—	0.089
Cramer’s V	—	—	0.071	—	0.014

A comparison of hospital campuses revealed that the antimicrobial usage rate at Dongjie Campus exceeded that at East City Campus in 2021 (21.58% vs 16.49%, χ² = 401.78, P < 0.001). However, this trend reversed starting in 2022 and persisted through 2024, with Dongjie Campus’s usage rate subsequently falling below that of East City Campus. Notably, the pathogen testing rate at Dongjie Campus remained significantly lower than that at East City Campus throughout the study period (2021: 7.05% vs 16.73%, χ² = 148.91, P < 0.001; 2024: 10.26% vs 15.04%, χ²=307.67, P<0.001) (See **Tables 3**, **4**; **Figure 1**).

**Table 3:** Antimicrobial use and pretreatment pathogen testing among emergency patients at two hospital campuses, 2021–2024.

Year	Dongjie campus					East city campus				
	Number of visits	Number of antibiotic users	Antibiotic use rate (%)	Number of pre-treatment tests	Pre-treatment testing rate (%)	Number of visits	Number of antibiotic users	Antibiotic use rate (%)	Number of pre-treatment tests	Pre-treatment testing rate (%)
2021	32,085	6,924	21.58	488	7.05	79,822	13,165	16.49	2,202	16.73
2022	61,089	9,235	15.12	798	8.64	72,559	11,304	15.58	2,113	18.69
2023	62,595	11,249	17.97	915	8.13	71,306	16,310	22.87	2,967	18.19
2024	69,589	14,368	20.65	1,474	10.26	85,329	20,633	24.18	3,103	15.04
*χ² (df=3)*	—	—	888.26	—	71.12	—	—	2810.19	—	96.34
*P (χ²)*	—	—	<0.001	—	<0.001	—	—	<0.001	—	<0.001
*Z*	—	—	8.34	—	7.41	—	—	47.85	—	−5.09
*P (Trend)*	—	—	7.48×10^-^¹^7^	—	1.30×10^-^¹³	—	—	<1×10^-^¹^6^	—	3.62×10^-7^
*Cramer’s V*	—	—	0.063	—	0.041	—	—	0.095	—	0.04

**Table 4:** Comparison of antimicrobial utilization rates and pathogen testing rates between campus I and campus II, 2021–2024.

Year	Indicator	Dongjie campus (%)	East city campus(%)	*χ²*	*P*
2021	Antibiotic Use Rate	21.58	16.49	401.78	<0.001
	Pathogen testing rate	7.05	16.73	148.91	<0.001
2022	Antibiotic Use Rate	15.12	15.58	5.4	0.020
	Pathogen testing rate	8.64	18.69	400.64	<0.001
2023	Antibiotic Use Rate	17.97	22.87	489.77	<0.001
	Pathogen testing rate	8.13	18.19	861.71	<0.001
2024	Antibiotic Use Rate	20.65	24.18	273.46	<0.001
	Pathogen testing rate	10.26	15.04	307.67	<0.001

### Distribution of pre-antibiotic therapy pathogen specimens in emergency patients across the two hospital campuses

**Figure 2:**
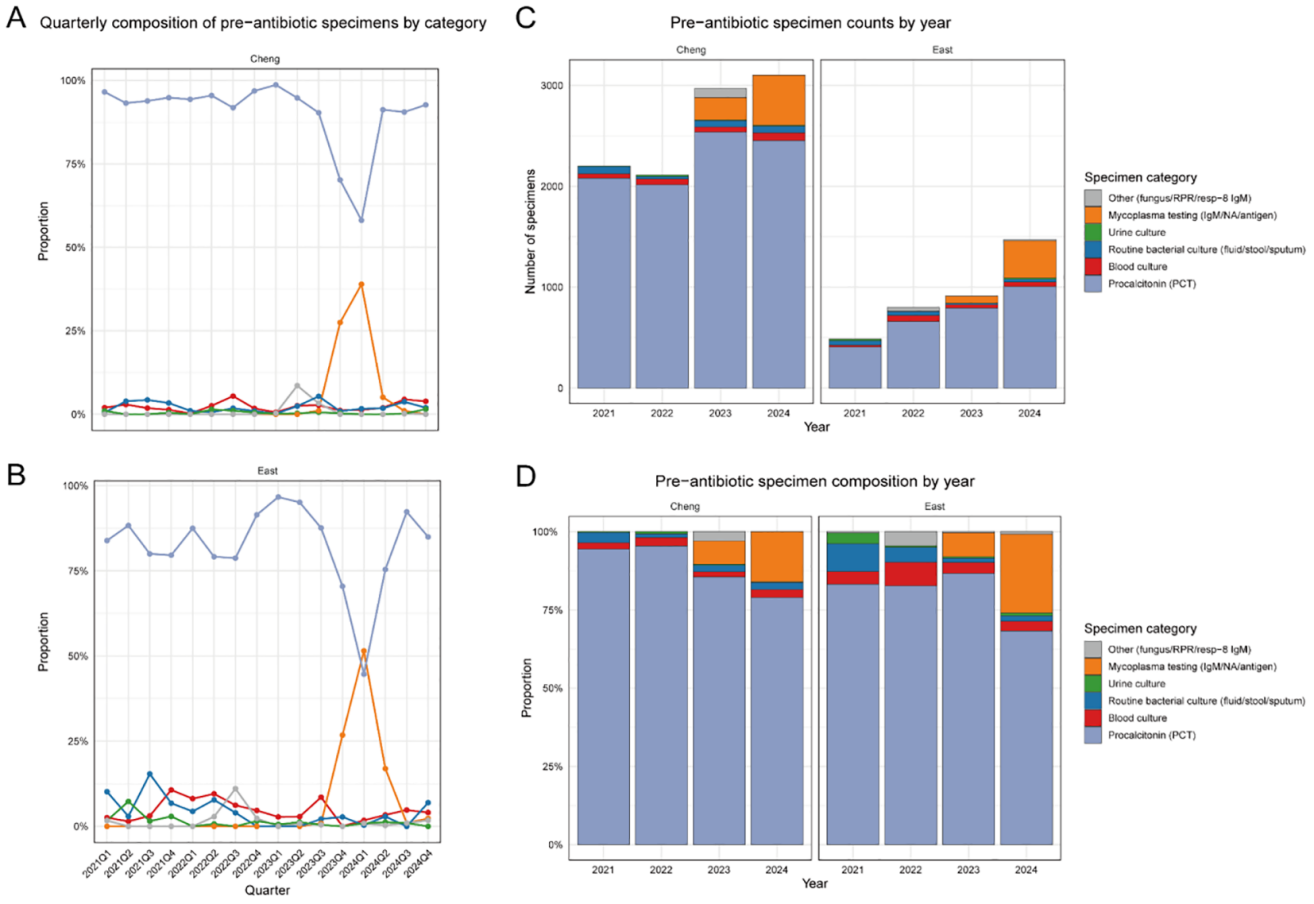
Preantibiotic microbiology specimens from emergency department patients at two sites (East vs Cheng), 2021–2024. **(A)** Quarterly composition, Cheng: lines/points show the proportion of each sample category in every quarter from 2021Q1–2024Q4. **(B)** Quarterly composition, East: same as **(A)** for the Dongjie Campus. **(C)** Annual counts: stacked bars by specimen category, faceted by site; the bar height equals the total number of preantibiotic samples in that year at the given site. **(D)** Annual composition: 100% stacked bars by specimen category; each bar sums to 100% for that year and site.

In terms of specimen composition, procalcitonin (PCT) consistently dominated, although its proportion declined at both campuses (Dongjie Campus: 83.20% → 68.25%, Z = −8.64, P < 0.001; East City Campus: 94.37% → 78.99%, Z = −19.54, P < 0.001). Mycoplasma pneumoniae-related testing has rapidly increased since 2023, accounting for 25.17% (371/1,474) of submitted items at Dongjie Campus and 15.98% (496/3,103) at East City Campus by 2024. Traditional bacterial cultures (blood, sputum, urine, etc.) consistently accounted for less than 5% of all tests (See **Table 5**; **Figure 2**).

**Table 5:** Distribution of infection-related laboratory tests in emergency patients across two hospital campuses before antimicrobial therapy, 2021–2024.

Dongjie Campus								
Item	2021	2022	2023	2024	Z	χ² (df=1)	P	Cramer’s V
Biomarkers								
Procalcitonin	406(83.20)	660(82.71)	793(86.67)	1006(68.25)	−8.64	74.65	<1e-16	0.143
Culture-based microbiology								
Blood Culture	20(4.10)	60(7.52)	32(3.50)	46(3.12)	−3.13	9.8	0.002	0.052
General Bacterial Culture (Aspirate, Feces, Cholera Culture, Sputum, etc.)	43(8.81)	38(4.76)	12(1.31)	26(1.76)	−7.70	59.29	1.40E-14	0.127
Urine Culture	17(3.48)	4(0.50)	5(0.55)	13(0.88)	−3.29	10.82	0.001	0.054
Serology/Antigen/Nucleic acid tests								
Mycoplasma pneumoniae IgM Antibody, MP Nucleic Acid, MP Antigen	0(0.00)	0(0.00)	70(7.65)	371(25.17)	19.33	373.65	<1e-16	0.319
Other								
Fungi, RPR, 8 Respiratory Pathogen IgM Antibodies, etc.	2(0.41)	36(4.51)	3(0.33)	12(0.81)	−3.06	9.36	0.002	0.051
Total	488(100.00)	798(100.00)	915(100.00)	1474(100.00)				
East City Campus								
Item	2021	2022	2023	2024	*Z*	*χ² (df=1)*	*P*	*Cramer’s V*
Biomarkers								
Procalcitonin	2078(94.37)	2016(94.51)	2538(85.54)	2451(78.99)	−19.54	381.81	<1e-16	0.192
Culture-based microbiology								
Blood Culture	46(2.09)	57(2.67)	50(1.69)	79(2.55)	0.37	0.14	0.709	0.004
General Bacterial Culture (Aspirate, Feces, Cholera Culture, Sputum, etc.)	72(3.27)	25(1.17)	62(2.09)	68(2.19)	−1.59	2.53	0.112	0.016
Urine Culture	6(0.27)	15(0.70)	9(0.30)	8(0.26)	−0.95	0.9	0.343	0.009
Serology/Antigen/Nucleic acid tests								
Mycoplasma pneumoniae IgM Antibody, MP Nucleic Acid, MP Antigen	0(0.00)	0(0.00)	221(7.45)	496(15.98)	25.41	645.67	<1e-16	0.249
Other								
Others (Fungi, RPR, 8 Respiratory Pathogen IgM Antibodies, etc.)	0(0.00)	0(0.00)	88(2.97)	1(0.03)	2.89	8.35	0.004	0.028
Total	2202(100.00)	2133(100.00)	2967(100.00)	3103(100.00)				

### Traditional bacterial culture submission rates per antibiotic-treated ED visit

Because proportions among submitted specimens may not reflect the actual intensity of culture testing among antibiotic-treated ED visits, we further calculated culture submission rates using antibiotic-treated ED visits as the denominator. Traditional bacterial cultures were defined as blood culture, routine bacterial culture, and urine culture. Overall, the combined traditional bacterial culture submission rate remained low throughout the study period: 1.02% in 2021, 0.97% in 2022, 0.62% in 2023, and 0.69% in 2024. When examined separately, blood culture rates ranged from 0.30% to 0.57%, routine bacterial culture rates from 0.27% to 0.57%, and urine culture rates from 0.05% to 0.11% across years. At the campus level, East showed a decrease in the combined traditional bacterial culture submission rate from 1.16% in 2021 to 0.59% in 2024, whereas Cheng remained below 1% in all years (0.94%, 0.86%, 0.74%, and 0.75% from 2021 to 2024). These findings confirm that traditional bacterial cultures were not only a small proportion of submitted specimens, but were also infrequently ordered among antibiotic-treated ED visits (**Table 6**).

**Table 6:** Traditional bacterial culture submission rates per antibiotic-treated ED visit, 2021-2024.

Campus	Year	Antibiotic-treated ED visits	Blood culture, n (%)	Routine bacterial culture, n (%)	Urine culture, n (%)	Combined traditional bacterial culture, n (%)
Overall	2021	20,089	66 (0.33)	115 (0.57)	23 (0.11)	204 (1.02)
	2022	20,539	117 (0.57)	63 (0.31)	19 (0.09)	199 (0.97)
	2023	27,559	82 (0.30)	74 (0.27)	14 (0.05)	170 (0.62)
	2024	35,001	125 (0.36)	94 (0.27)	21 (0.06)	240 (0.69)
Dongjie Campus	2021	6,924	20 (0.29)	43 (0.62)	17 (0.25)	80 (1.16)
	2022	9,235	60 (0.65)	38 (0.41)	4 (0.04)	102 (1.10)
	2023	11,249	32 (0.28)	12 (0.11)	5 (0.04)	49 (0.44)
	2024	14,368	46 (0.32)	26 (0.18)	13 (0.09)	85 (0.59)
East City Campus	2021	13,165	46 (0.35)	72 (0.55)	6 (0.05)	124 (0.94)
	2022	11,304	57 (0.50)	25 (0.22)	15 (0.13)	97 (0.86)
	2023	16,310	50 (0.31)	62 (0.38)	9 (0.06)	121 (0.74)
	2024	20,633	79 (0.38)	68 (0.33)	8 (0.04)	155 (0.75)

### Preantimicrobial therapy pathogen testing rates in emergency internal medicine and surgery groups across both campuses

**Figure 3:**
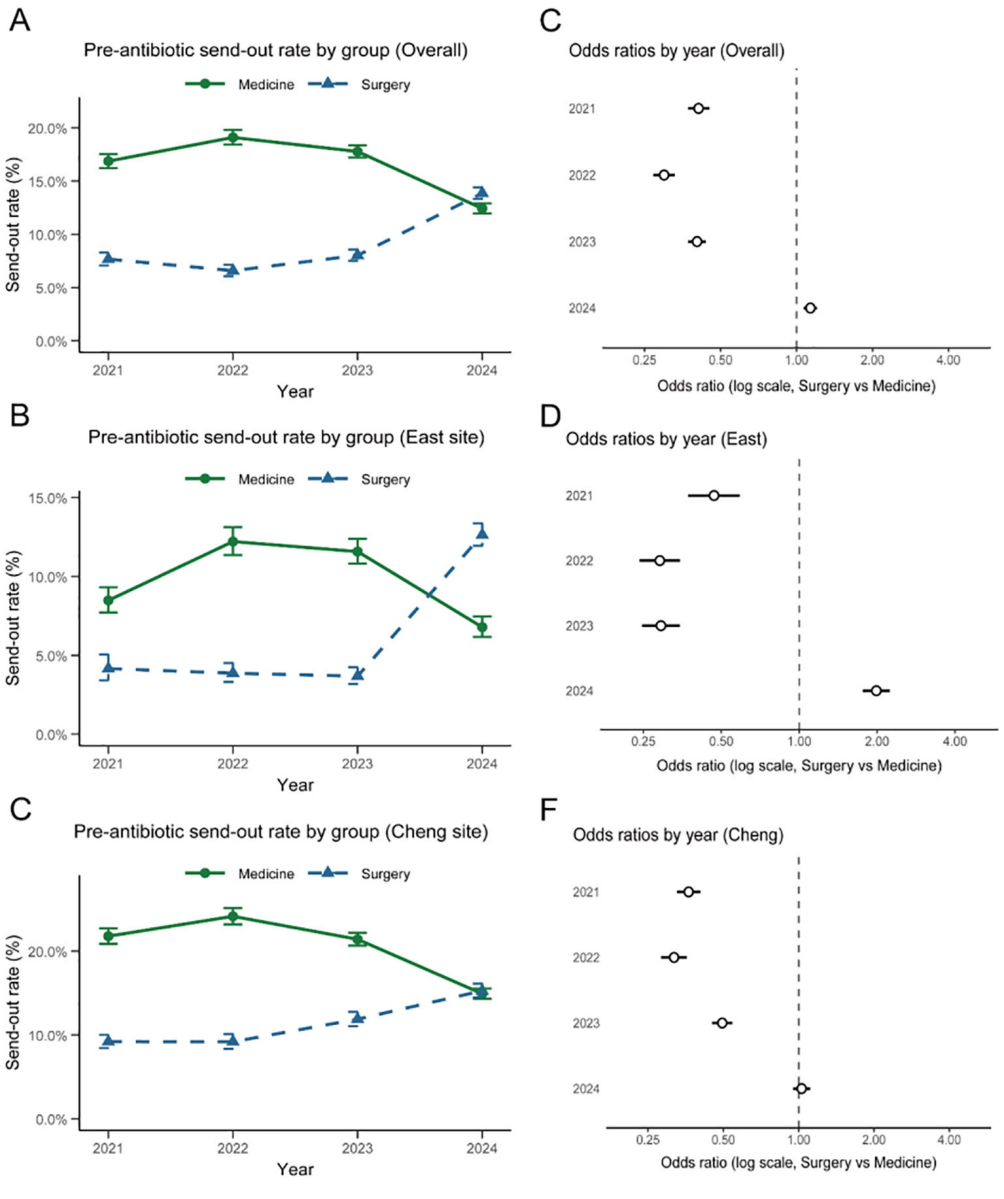
Preantibiotic microbiology is sent out in emergency departments from 2021–2024. **(A–C)** Yearly send-out rate (%) with 95% Wilson confidence intervals for Medicine (solid green) and Surgery (dashed blue): **(A)** Overall cohort, **(B)** Dongjie Campus, **(C)** East City Campus. **(D–F)** Odds ratios (surgery vs medicine) by year with 95% confidence intervals on a log scale; the vertical dashed line marks OR = 1 (no difference): **(D)** overall, **(E)** east, **(F)** Cheng.

There were significant differences in preantimicrobial therapy pathogen testing rates between the emergency internal medicine and surgery groups. From 2021 to 2023, the submission rate in the medical department was significantly higher than that in the surgical department (all P < 0.001). However, this trend reversed for the first time in 2024 (12.42% in medical departments vs. 13.86% in surgical departments, χ² = 15.72, P < 0.001). In addition, Cramer’s V = 0.073 for internal medicine and 0.105 for surgery, indicating that although the difference was significant, the effect size was small (See **Table 7**; **Figure 3**). The surgical submission rate at Dongjie Campus increased from 4.16% in 2021 to 12.64% in 2024 (trend χ² = 633.22, P < 0.001), whereas internal medicine decreased from 8.49% to 6.79% (trend χ² = 127.70, P < 0.001), but Cramer’s V = 0.098, indicating that although the difference was significant, the effect size was small (See **Table 8**; **Figure 3**). The East City Campus also showed an increasing trend in surgery (9.19% → 15.26%, trend χ² = 89.10, P < 0.001), whereas internal medicine decreased from 21.78% to 14.91% (trend χ² = 83.97, P < 0.001), but Cramer’s V = 0.07, indicating that although the difference was significant, the effect size was small (See **Table 9**; **Figure 3**).

**Table 7:** Comparison of pre-antimicrobial therapy pathogen testing rates between emergency internal medicine and surgery groups.

Year	Medical group			Surgical group			*χ²*	*P*	*Cramer’s V*
	Number of antibiotic Users	Number of tests	pathogen testing rate(%)	Number of antibiotic Users	Number of tests	pathogen testing rate (%)			
2021	12,497	2,108	16.87	7,592	582	7.67	344.839	<0.001	0.131
2022	12,460	2,379	19.09	8,079	532	6.58	630.368	<0.001	0.175
2023	17,137	3,046	17.77	10,422	836	8.02	509.373	<0.001	0.136
2024	19,076	2,370	12.42	15,925	2,207	13.86	15.718	<0.001	0.021
Z			−12.31			10.68			
χ²			151.61			114.12			
P			<1e−16			<1e−16			
Cramer’s V			0.073			0.105			

**Table 8:** Comparison of test submission rates between Internal medicine and surgery teams in the Dongjie campus emergency department.

Year	Medical group			Surgical group			*χ²*	*P*	*Cramer’s V*
	Number of antibiotic users	Number of tests	Pathogen testing rate(%)	Number of antibiotic users	Number of tests	Pathogen testing rate (%)			
2021	4,619	392	8.49	2,305	96	4.16	43.84	<0.001	0.08
2022	5,281	645	12.21	3,954	153	3.87	199.415	<0.001	0.147
2023	6,339	734	11.58	4,910	181	3.69	230.666	<0.001	0.143
2024	5,846	397	6.79	8,522	1,077	12.64	128.755	<0.001	0.095
*Z*			−11.30			25.16			
*χ²*			127.7			633.22			
*P*			<1e−16			<1e−16			
*Cramer’s V*			0.098			0.237			

**Table 9:** Comparison of test submission rates between emergency internal medicine and emergency surgery teams at East City campus.

Year	Medical group			Surgical group			*χ²*	*P*	*Cramer’s V*
	Number of Antibiotic Users	Number of Tests	Pathogen testing rate (%)	Number of Antibiotic Users	Number of Tests	Pathogen testing rate (%)			
2021	7,878	1,716	21.78	5,287	486	9.19	360.03	<0.001	0.165
2022	7,179	1,734	24.15	4,125	379	9.19	386.068	<0.001	0.185
2023	10,798	2,312	21.41	5,512	655	11.88	222.617	<0.001	0.117
2024	13,230	1,973	14.91	7,403	1,130	15.26	0.458	0.499	0.005
*Z*			−9.16			9.44			
*χ²*			83.97			89.1			
*P*			<1e−16			<1e−16			
*Cramer’s V*			0.07			0.105			

## Discussion

This study systematically monitored over 530,000 emergency department visits across two campuses of a tertiary hospital from 2021--2024, revealing the current status, trends, and issues surrounding emergency antimicrobial use and pretreatment pathogen testing. Key findings include a significant upward trend in antimicrobial usage rates, whereas pathogen testing rates remained persistently low (13%-14%), with notable variations across campuses and departments (internal/surgical). The test samples were predominantly procalcitonin (PCT)-based, although the proportion of PCT-based samples declined annually. Testing for Mycoplasma pneumoniae has rapidly increased, whereas traditional microbial culture testing rates have remained extremely low (<5%). These findings not only confirm the severe challenges in managing emergency antimicrobial use in this region but also provide clear direction and robust data support for subsequent targeted interventions.

Over a four-year period, the rate of antimicrobial use in emergency departments rose from 17.95% to 22.59% (Z = 41.79, p<0.001). This rising trend in antimicrobial use presents a challenge to national goals of curbing bacterial resistance and promoting rational drug use, highlighting stewardship challenges. This may be due to doctors increasingly relying on empirical broad-spectrum antibiotics when treating patients with complex, critical conditions and unknown causes of infection in emergency settings.In stark contrast, pretreatment pathogen testing rates did not improve (P = 0.089), fluctuating persistently at low levels between 13% and 14%. This scissors gap phenomenon of “high drug use, low testing” constitutes the core contradiction in current emergency infection diagnosis and treatment. Low testing rates suggest that a substantial proportion of antimicrobial use may not be guided by microbiological evidence. This not only hinders precision treatment and improved patient outcomes but also intensifies selective pressure on antimicrobial agents, creating significant risks for the proliferation and spread of drug-resistant bacteria (Timbrook et al., 2017) (Boracchini et al., 2024). Our findings highlight the urgency and central importance of increasing testing rates in emergency antimicrobial stewardship (AMS).

The two hospital campuses exhibit significant and intriguing differences in antimicrobial usage rates and pathogen testing rates. Campus One’s antimicrobial usage rate shifted from being higher than that of Campus Two’s to being lower, yet its pathogen testing rate consistently lagged significantly behind. The persistent difference in testing rates between the two campuses likely reflects structural differences in various aspects, including hardware facilities, patient population, management and operational models, laboratory service capabilities, testing prescription procedures, and the rigor of antibiotic prescription review.For example, East City Campus have a more convenient testing process or a stricter prescription review system. Future research needs to delve deeper into the impact of these specific factors on testing behavior.

A more profound finding lies in the differences and dynamic shifts in referral patterns between internal medicine and surgery departments. From 2021 to 2023, internal medicine departments presented significantly higher referral rates than surgical departments did. This disparity stems from internal medicine physicians’ greater involvement in managing suspected bacterial infections affecting the respiratory and urinary systems, whereas surgeons maintain a mindset focused on perioperative prophylaxis and posttraumatic management (Zeng et al., 2022). However, 2024 data showed a reversal, with surgical departments surpassing medical departments in pathogen testing rates for the first time (13.86% vs. 12.42%). Both campuses presented a significant upward trend in surgical pathogen testing rates, whereas medical pathogen testing rates declined markedly. This shift may stem from the initial effectiveness of targeted management measures for surgical departments (e.g., stricter testing requirements for perioperative prophylactic antibiotics) while also signaling potential laxity or emerging barriers in testing management for medical departments (e.g., the Mycoplasma pneumoniae outbreak may have altered testing patterns). This dynamic divergence underscores the necessity for refined, department-specific management strategies.

Analysis of the composition of submitted samples reveals another deep-seated issue in current emergency testing practices: heavy reliance on inflammatory markers (particularly PCT) while neglecting traditional microbial culture. Although indicators such as PCT hold significant value in distinguishing bacterial infections and guiding the discontinuation of antimicrobial therapy (Schuetz et al., 2018), they provide indirect evidence and cannot replace the direct evidence of pathogen identification and resistance provided by microbial culture and susceptibility testing. This study indicates that while PCT testing accounts for the greatest proportion of tests, it shows a declining trend, whereas Mycoplasma pneumoniae testing has surged due to recent outbreaks—both falling under “nonculture” testing. The proportion of bacterial cultures submitted for blood, sputum, urine, and other samples—which provide the “gold standard” for pathogen diagnosis—remains persistently below 5%, a concerning phenomenon. This reflects a potential tendency in clinical practice to prioritize rapid screening over confirmatory cultures. The contributing factors may include the time-consuming nature of cultures, stringent specimen collection requirements, cumbersome submission processes, and the impact of prior antibiotic use on positive rates (Lalezari et al., 2020) (Dempsey et al., 2019) (Nauclér et al., 2021). This imbalance in testing patterns severely limits the implementation of targeted antimicrobial therapy and the early detection of drug-resistant bacteria, rendering antimicrobial stewardship (AMS) and infection control efforts akin to “viewing flowers through a haze.

Clinical Decision Support (CDS) systems based on information technology can improve the current situation of “high antibiotic use and low pathogen testing rate,” but there are still some obstacles to the implementation of this system. Firstly, technical challenges, such as the lack of seamless integration between existing Electronic Medical Record (EMR) platforms and CDS modules, often hinder the provision of real-time decision support services. Secondly, issues related to data quality and standardization, such as incomplete or unstructured laboratory and microbiology data, limit the effectiveness of algorithm-driven recommendations. Thirdly, organizational factors, including inadequate governance structures, limited financial resources for system maintenance, and insufficient training for clinicians, contribute to low adoption rates. To successfully deploy CDS to bridge the identified gaps, addressing these obstacles is crucial.

In summary, on the basis of the findings of this study, the following recommendations are proposed: (1) Multidisciplinary Collaborative Management Model: The infection control department, in collaboration with the Medical Affairs Department, Nursing Department, Laboratory Department, Pharmacy Department, and Information Technology Department, implements a series of targeted measures to strengthen the management of preantimicrobial pathogen testing in the Emergency Department. The Medical Affairs Department should intensify training and supervision on rational antimicrobial use among physicians. The Nursing Department should monitor whether nursing staff collect and submit relevant pathogen specimens in a timely and standardized manner. The Microbiology Laboratory of the Laboratory Department should ensure specimen quality control, requesting that physicians resubmit specimens that fail to meet standards. The Pharmacy Department should strictly control the indications for using key monitored, second-line, and third-line antimicrobials. The Information Technology Department utilizes digital interventions: the information system automatically prompts a pop-up reminder for pathogen testing when prescribing restricted-use antimicrobial agents and mandates a pathogen testing order before prescribing special-use antimicrobial agents (Helou et al., 2020). The infection control department monitors data, conducts training, identifies issues, and provides timely feedback to departments for rectification. Each department is responsible for implementing measures and pursuing continuous quality improvement. (2) Fully leveraging the antimicrobial stewardship (AMS) team: Apply the PDCA cycle management method to analyze causes, define improvement goals, formulate corrective actions, and regularly track outcomes. Antimicrobial stewardship training, case discussions, and feedback sessions should be integrated. (3) Establishing an evaluation system: Developing a data-driven evaluation framework incorporating process and outcome indicators to ensure evidence-based decision-making. This enables rational antimicrobial use, reduces the denominator of usage rates, and ensures that all tests that should be performed are submitted, thereby increasing the numerator of the pathogen testing rate. (4) Establish a long-term supervision and incentive mechanism: Continuously provide targeted education and training for medical staff at the hospital level, particularly for emergency surgery personnel. The information technology infrastructure should be strengthened, and a sustainable supervision and incentive system should be established to ensure continuous improvement in pathogen testing rates and the rational use of antimicrobial agents.

## Conclusions

This study, which is based on a large sample of real-world data collected over four consecutive years, confirms a significant imbalance between antimicrobial use in the emergency department and pathogen testing at this hospital, with complex influencing factors. Antimicrobial usage rates have risen annually, whereas pathogen testing rates have not increased synchronously, suggesting that current antimicrobial stewardship (AMS) strategies lack specificity and enforceability in emergency settings. Differences between departments and between internal medicine and surgery indicate the need for refined, tiered management. Specimen composition for testing relies excessively on inflammatory markers while traditional cultures are neglected, severely hindering subsequent targeted therapy and resistance monitoring. Based on our findings, we recommend focusing on multidisciplinary collaboration, refined assessment methods, and the application of information technology interventions. These measures may help improve pathogen testing rates and promote the rational use of antimicrobial drugs.

## Data Availability

The raw data supporting the conclusions of this article will be made available by the authors, without undue reservation.
